# Supplementary figures and images for: Transcriptional inhibition after irradiation occurs preferentially at highly expressed genes in a manner dependent on cell cycle progression
Source: eLife. 2024 Oct 11;13:RP94001. doi: 10.7554/eLife.94001 (PMC11469672; doi:10.7554/eLife.94001)

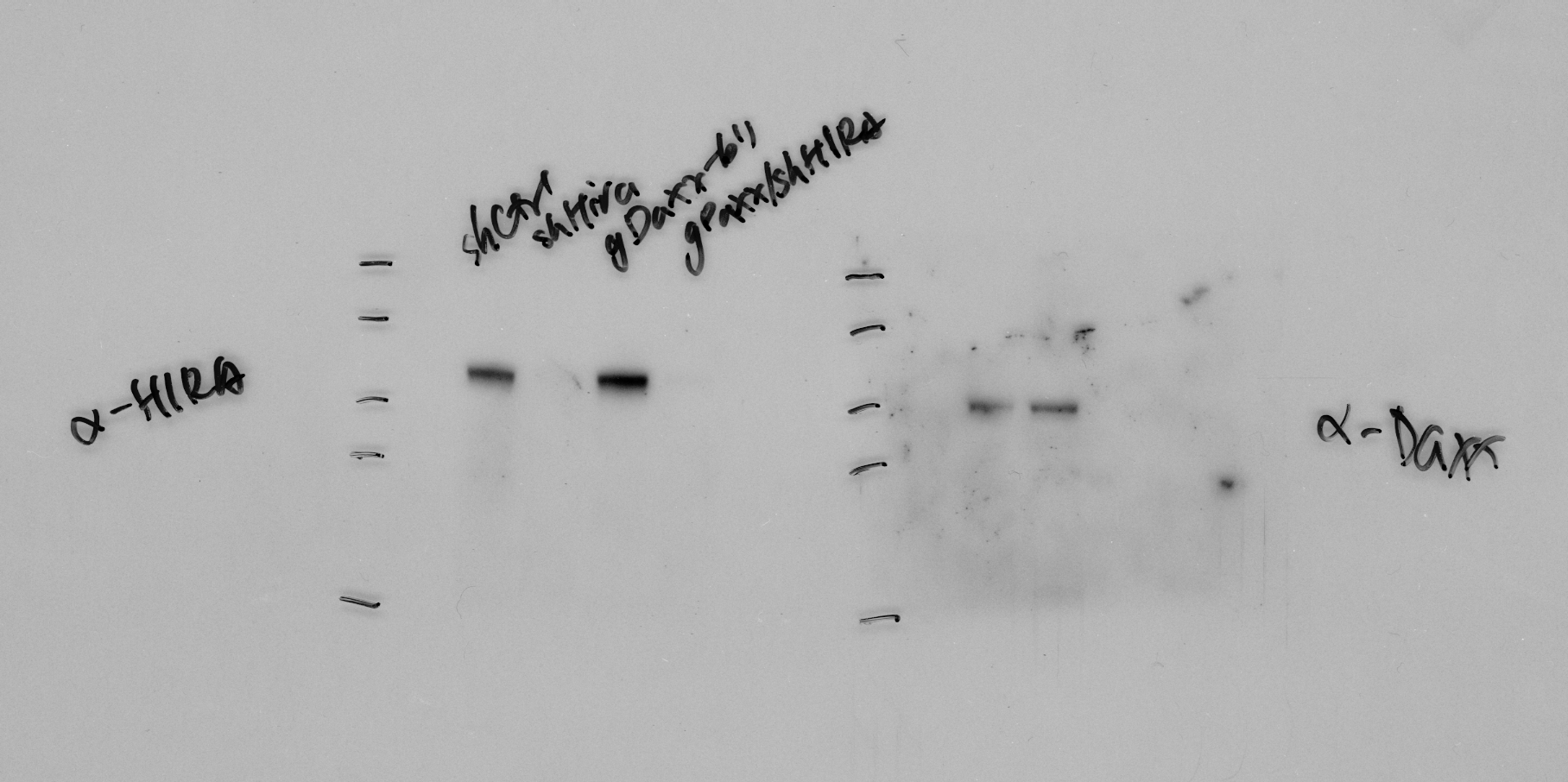

Supplement: Figure 1—figure supplement 2—source data 1. [file elife-94001-fig1-figsupp2-data1.zip › Figure 1-figure supplement 2 source data 2 HIRA western.tif]

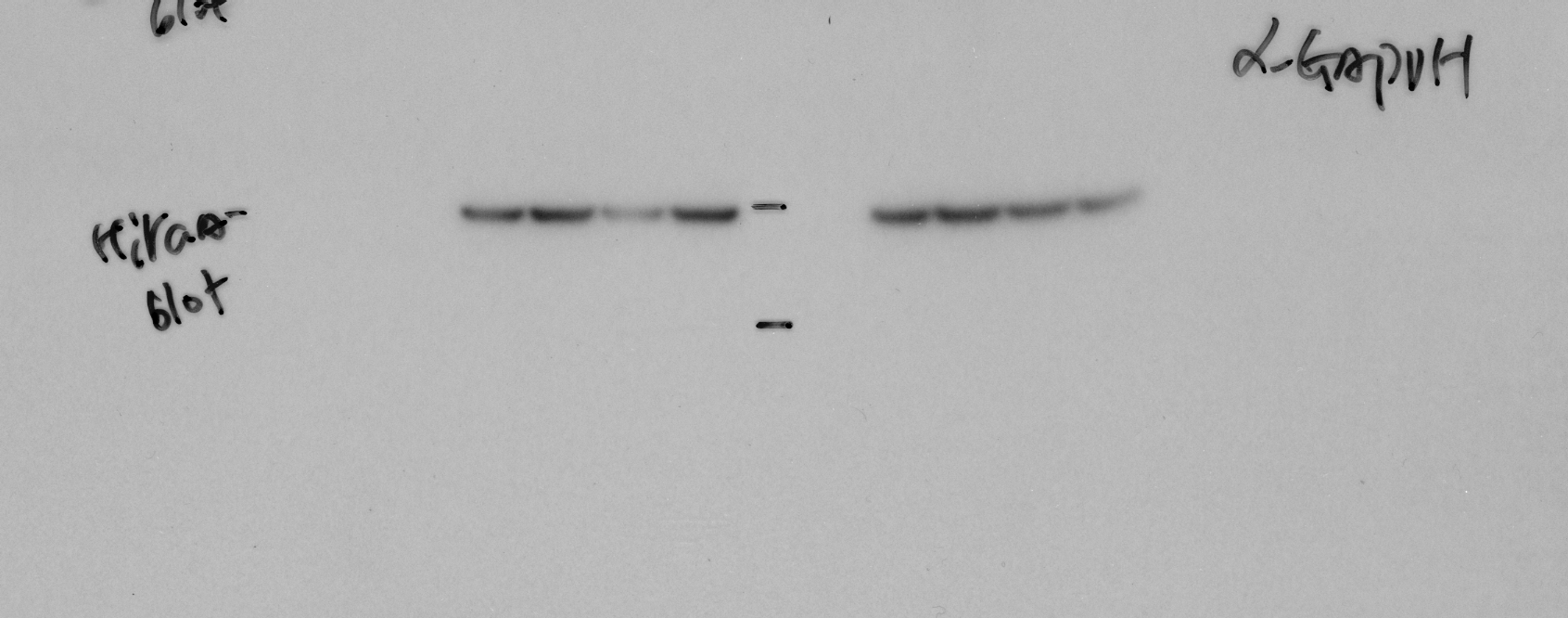

Supplement: Figure 1—figure supplement 2—source data 1. [file elife-94001-fig1-figsupp2-data1.zip › Figure 1-figure supplement 2 source data GAPDH for HIRA western.tif]

Figure 1-figure supplement 2 source data 2

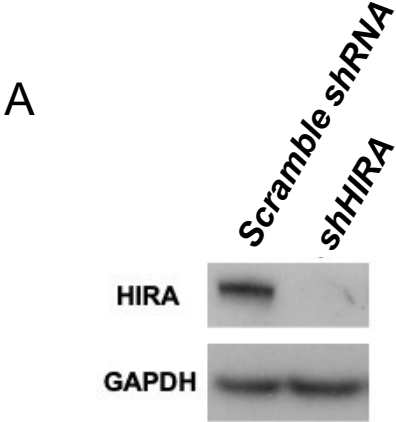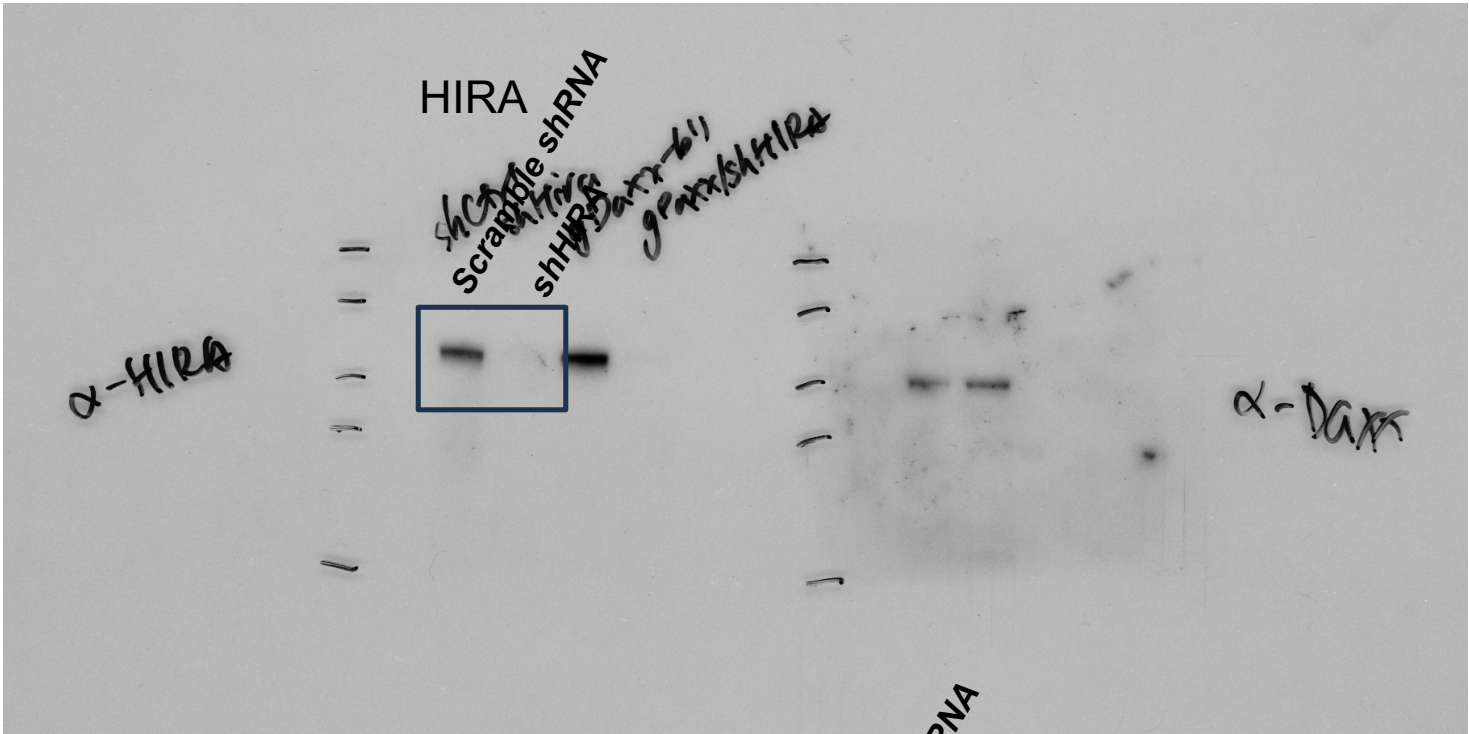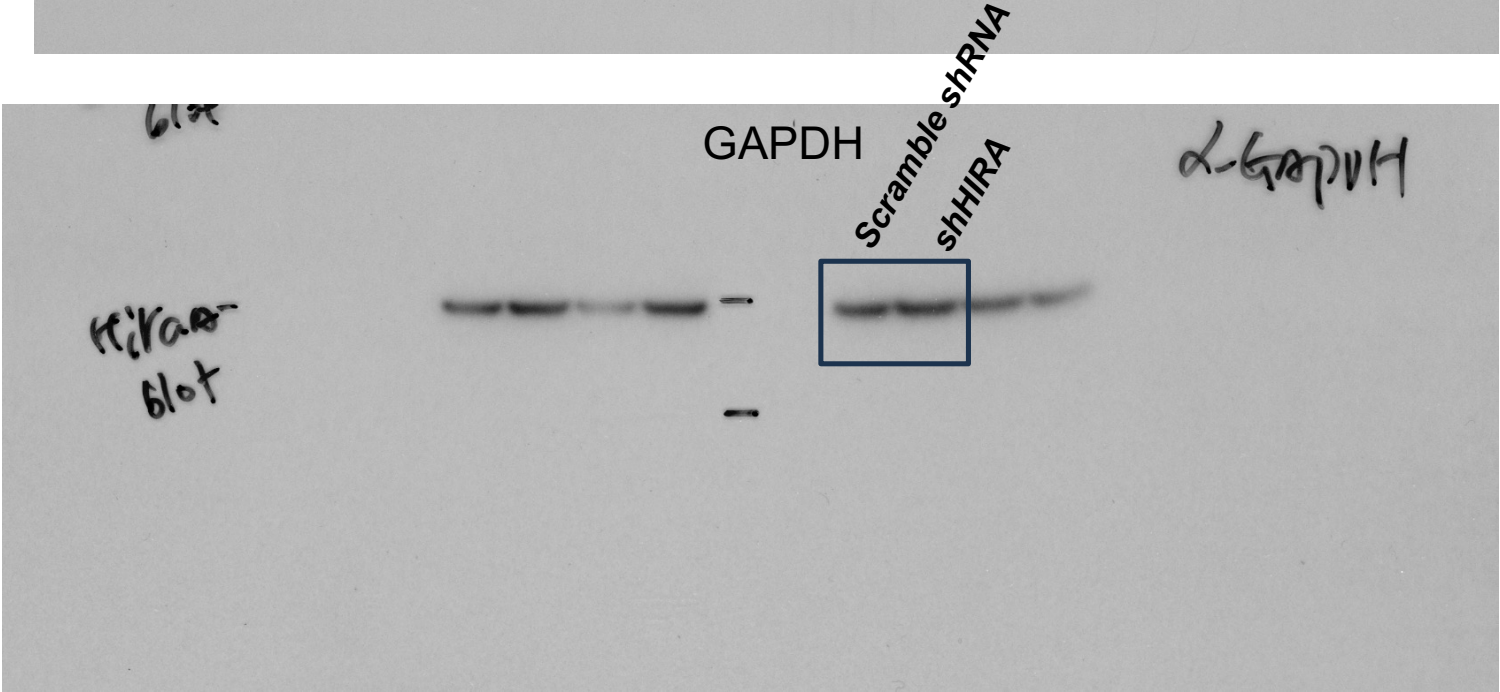

Supplement: Figure 1—figure supplement 2—source data 2. [file elife-94001-fig1-figsupp2-data2.pdf]

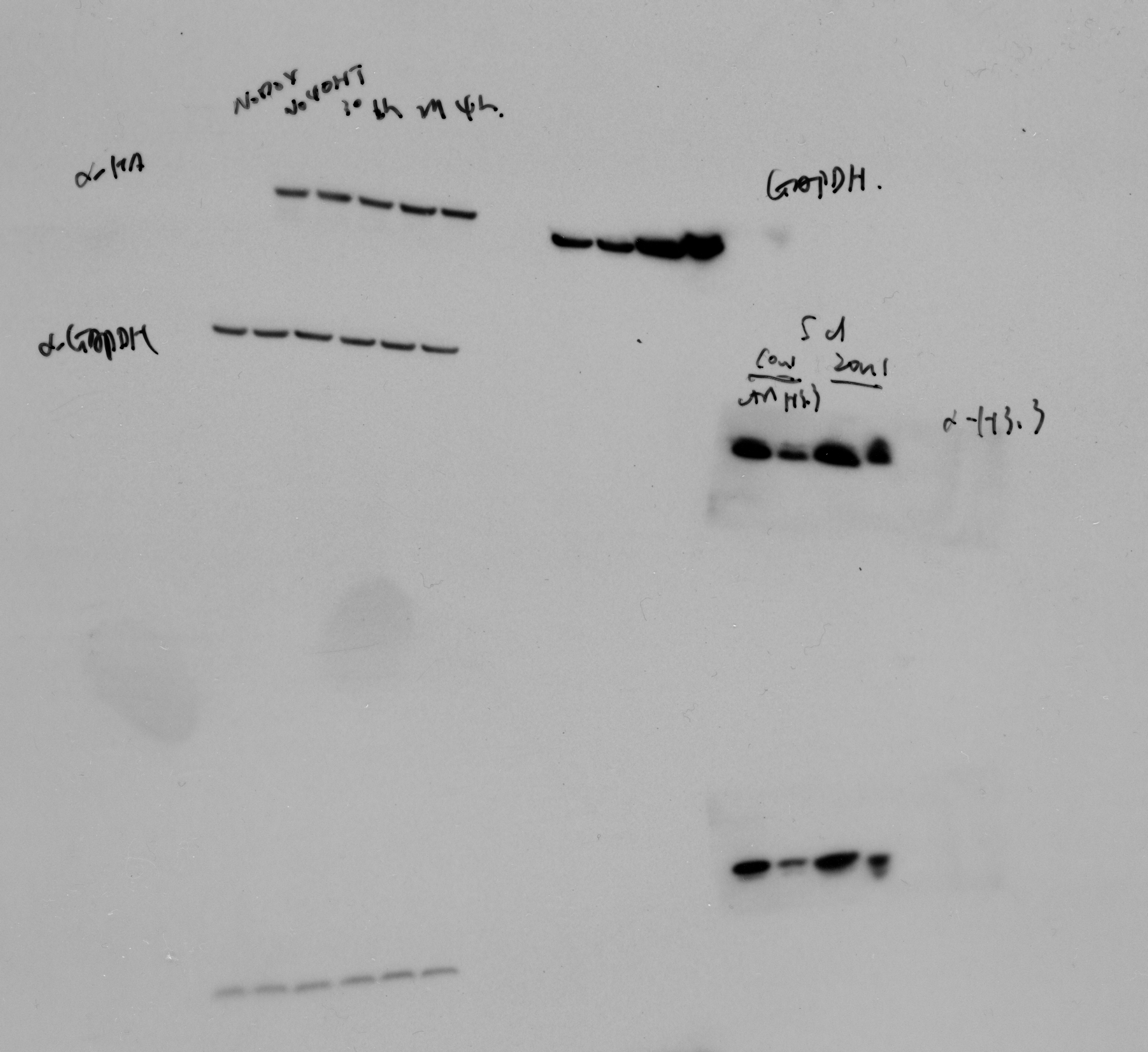

Supplement: Figure 1—figure supplement 2—source data 3. [file elife-94001-fig1-figsupp2-data3.tif]

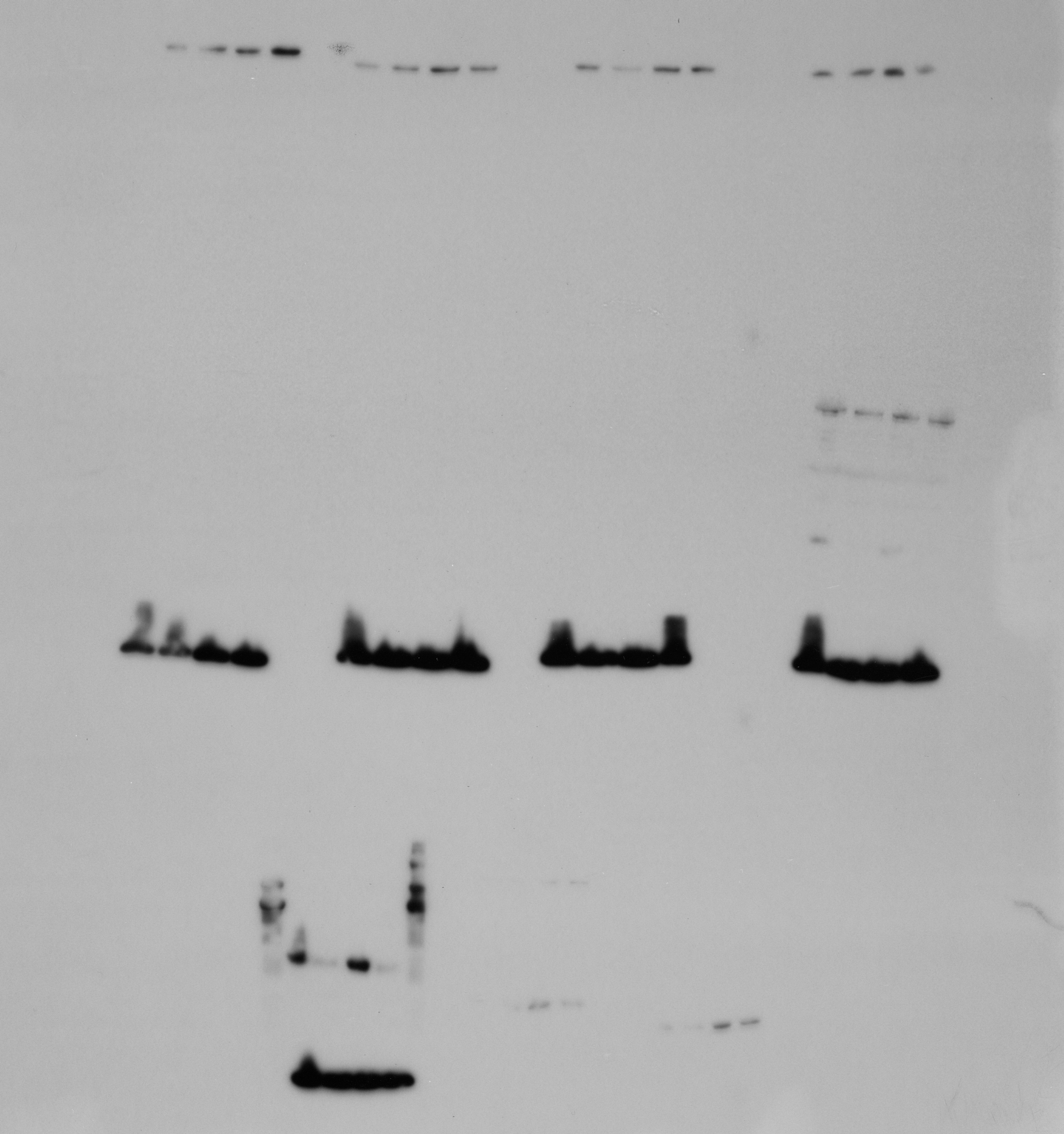

Supplement: Figure 5—figure supplement 2—source data 1. [file elife-94001-fig5-figsupp2-data1.zip › Figure 5-figure supplement 2 source data GAPDH for NAE1 western.tif]

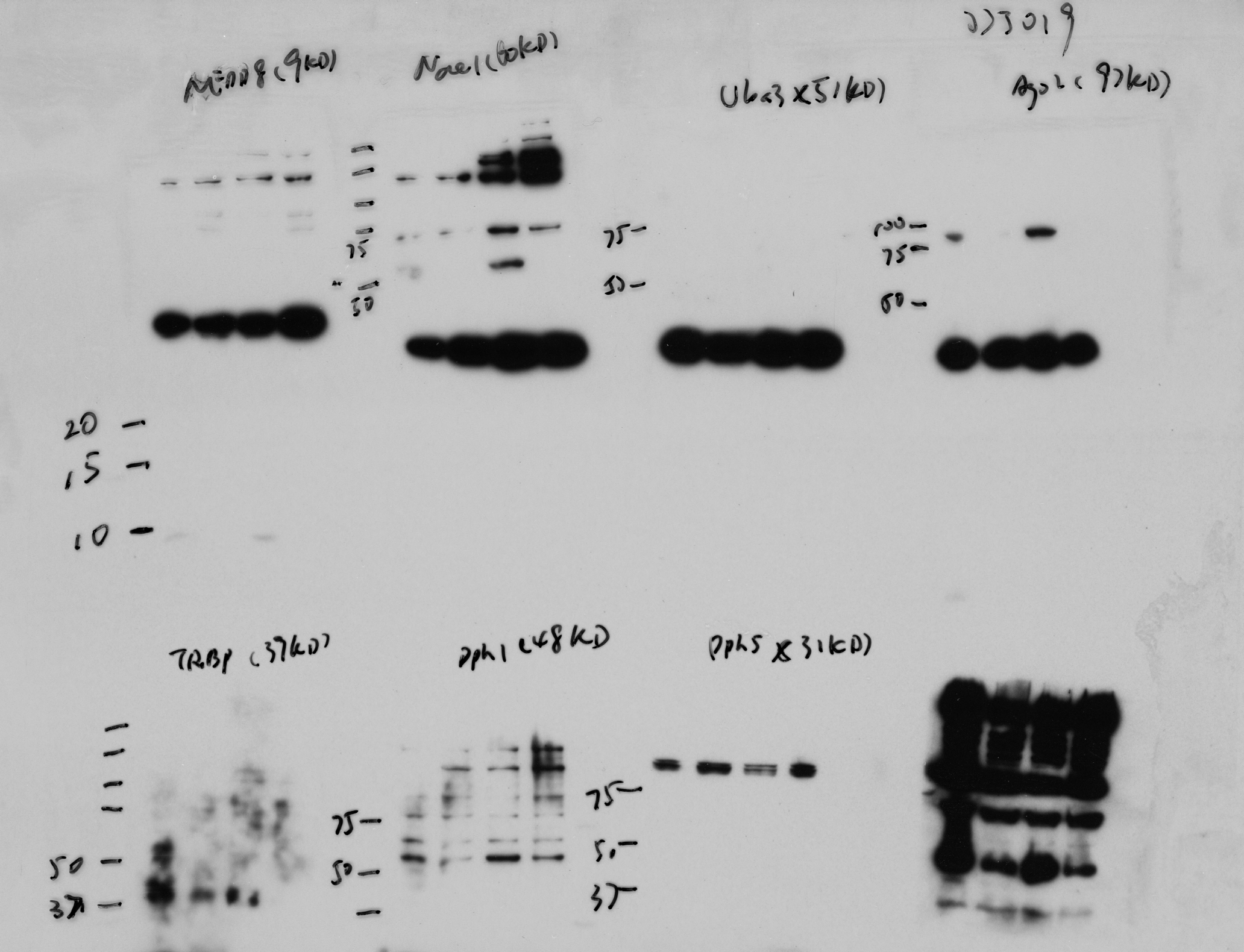

Supplement: Figure 5—figure supplement 2—source data 1. [file elife-94001-fig5-figsupp2-data1.zip › Figure 5-figure supplement 2 source data NAE1 western.tif]

Figure 5-figure supplement 2 –source data 2

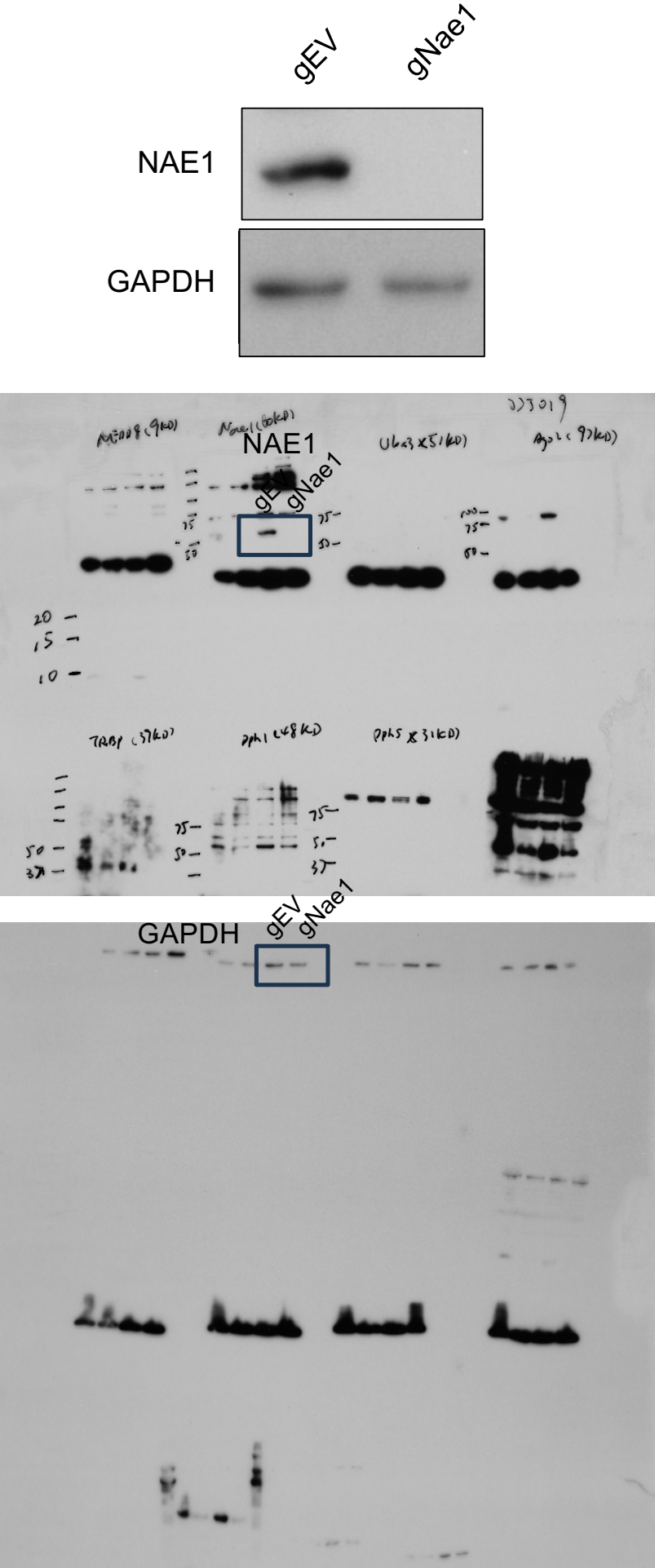

Supplement: Figure 5—figure supplement 2—source data 2. [file elife-94001-fig5-figsupp2-data2.pdf]

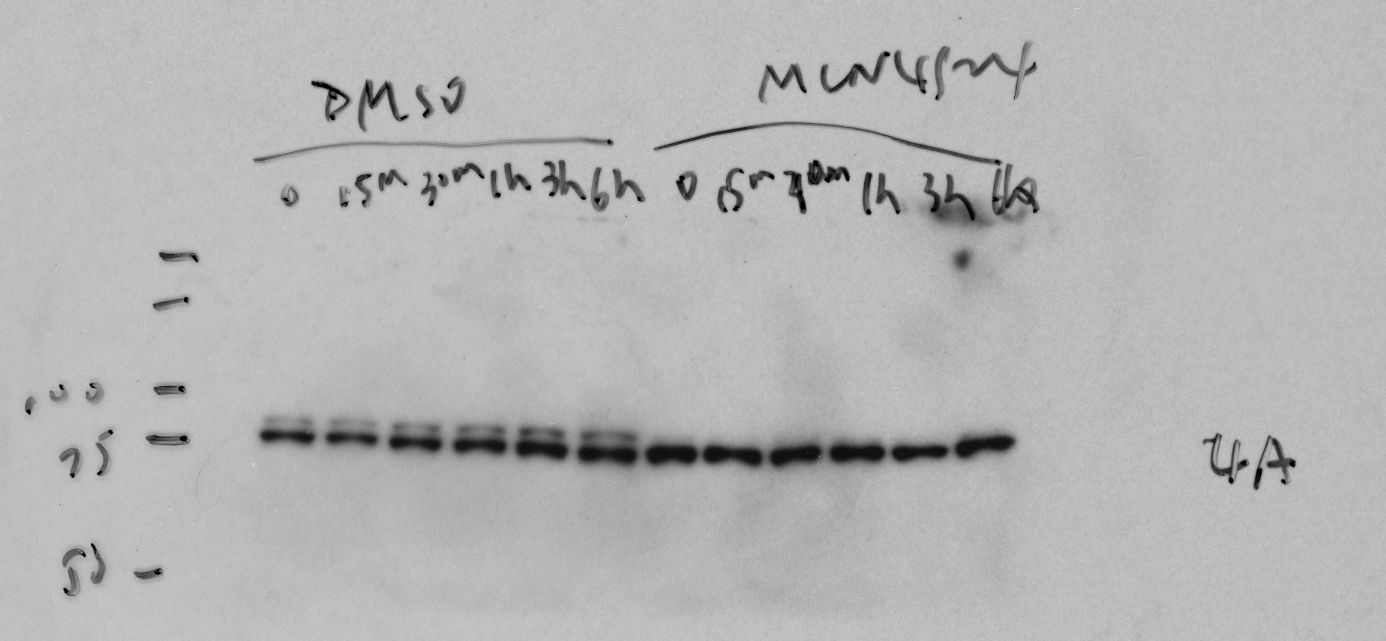

Supplement: Figure 6—source data 1. [file elife-94001-fig6-data1.zip › Figure 6 source data Cul4A western for panel A.tif]

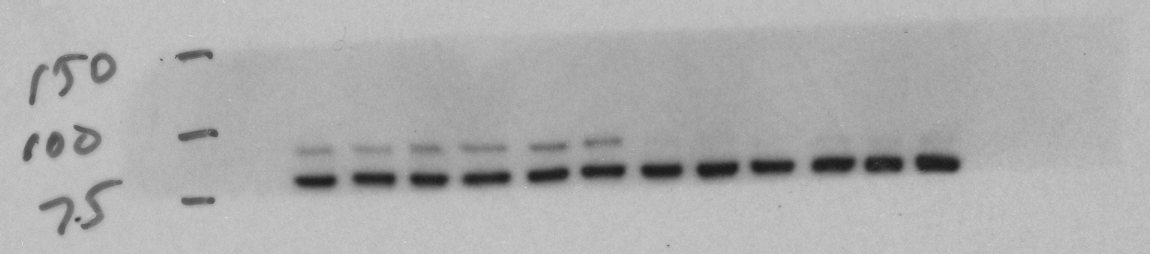

Supplement: Figure 6—source data 1. [file elife-94001-fig6-data1.zip › Figure 6 source data Cul4B western for panel A.tif]

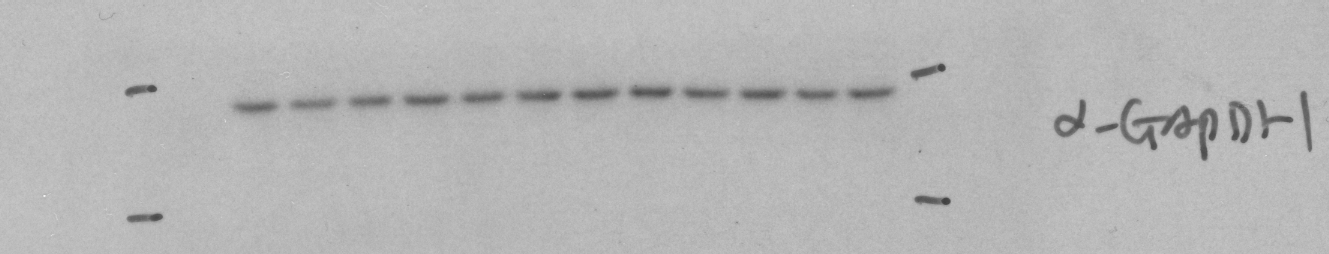

Supplement: Figure 6—source data 1. [file elife-94001-fig6-data1.zip › Figure 6 source data GAPDH western for panel A.tif]

Figure 6 Source data 2

A

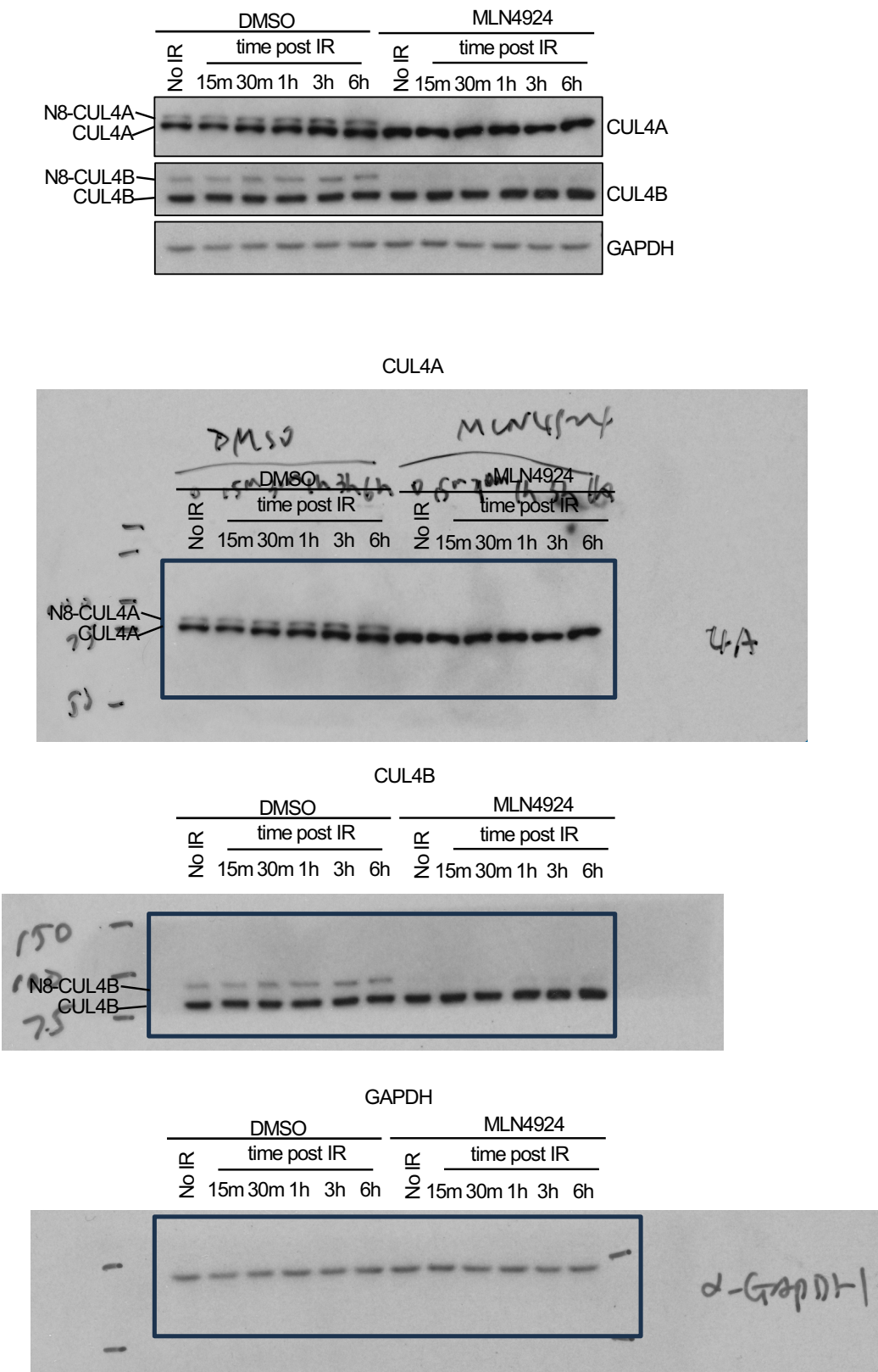

Supplement: Figure 6—source data 2. [file elife-94001-fig6-data2.pdf]

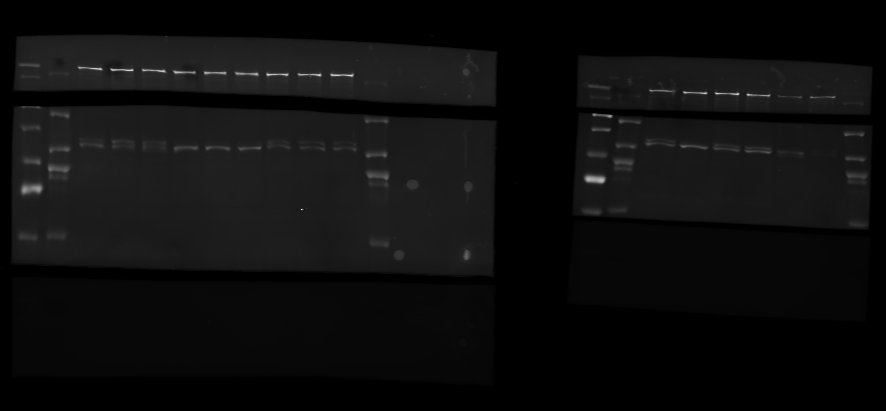

Supplement: Figure 6—source data 3. [file elife-94001-fig6-data3.zip › Figure 6 source data 1 Cul4B western for panel C.jpg]

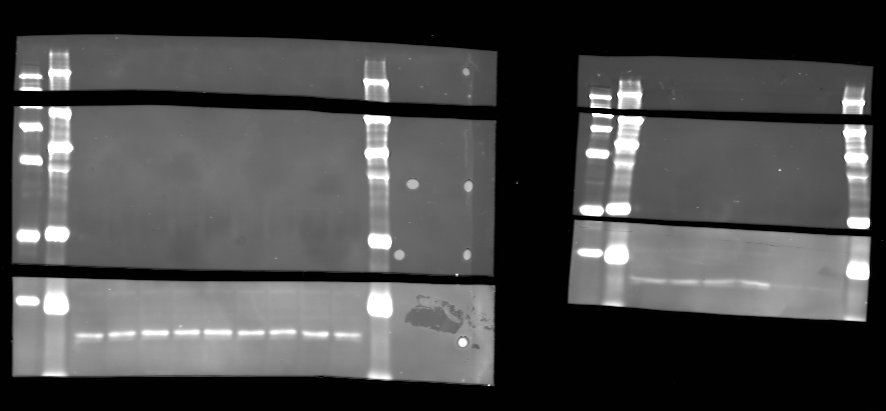

Supplement: Figure 6—source data 3. [file elife-94001-fig6-data3.zip › Figure 6 source data 1 GAPDH western for panel C.jpg]

Figure 6 Source data 4

C

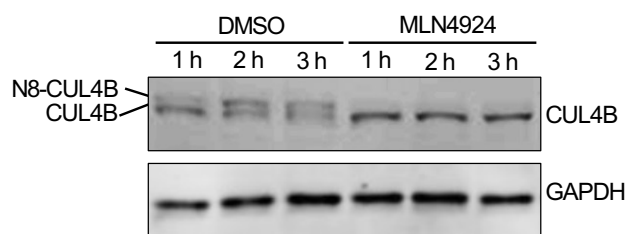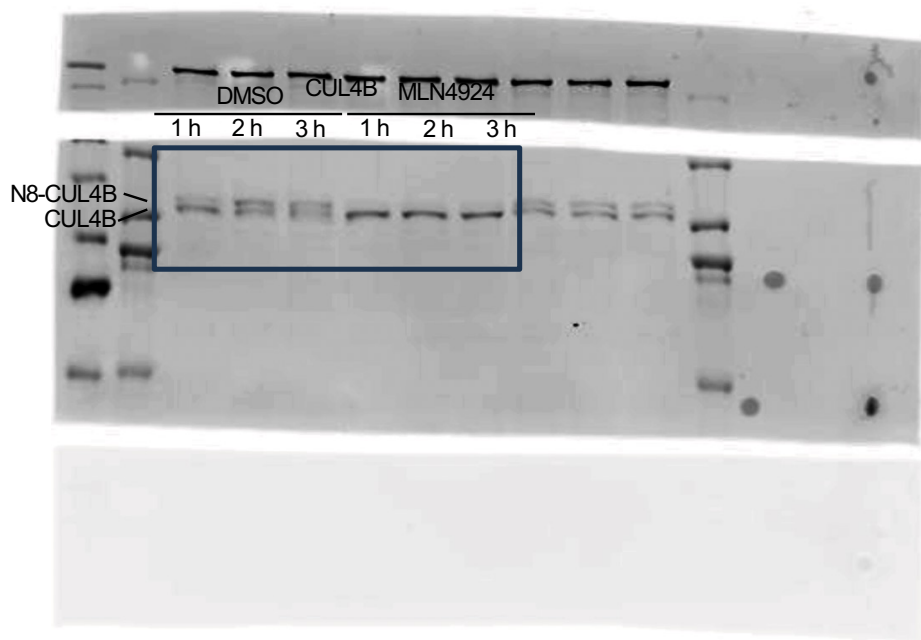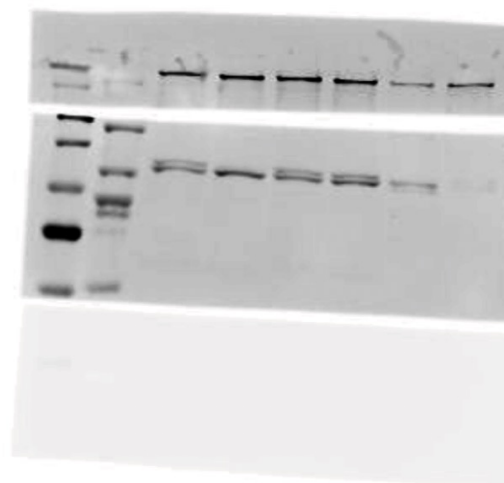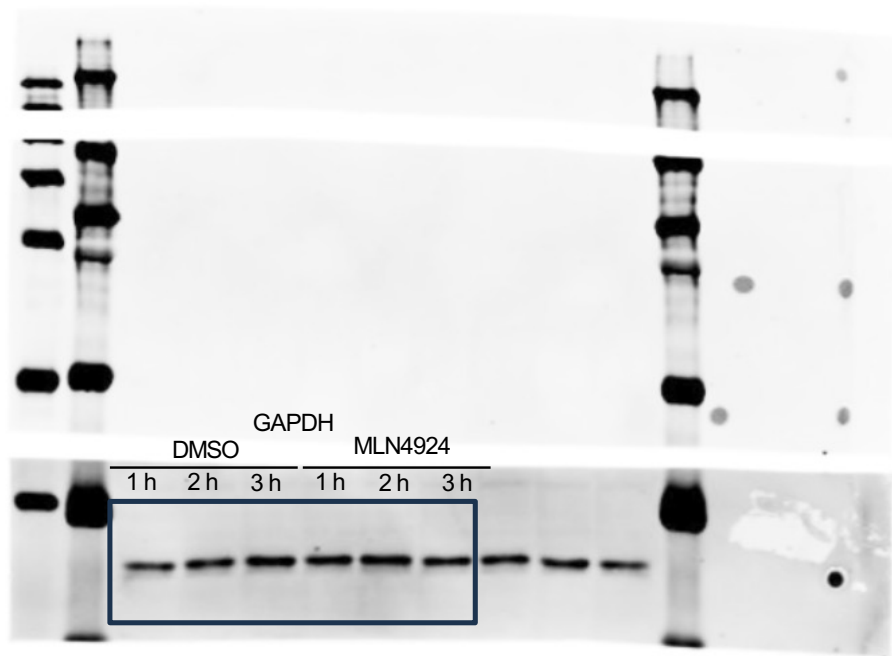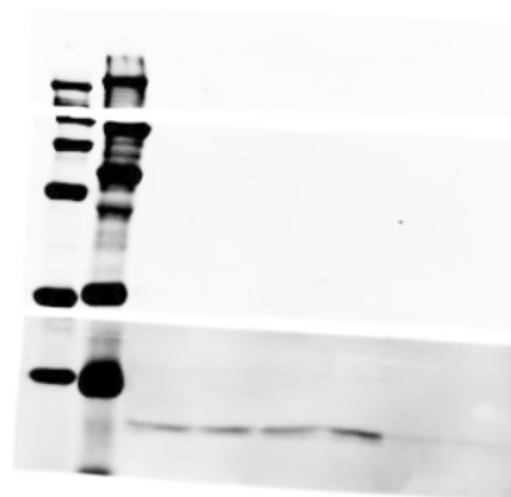

Supplement: Figure 6—source data 4. [file elife-94001-fig6-data4.pdf]

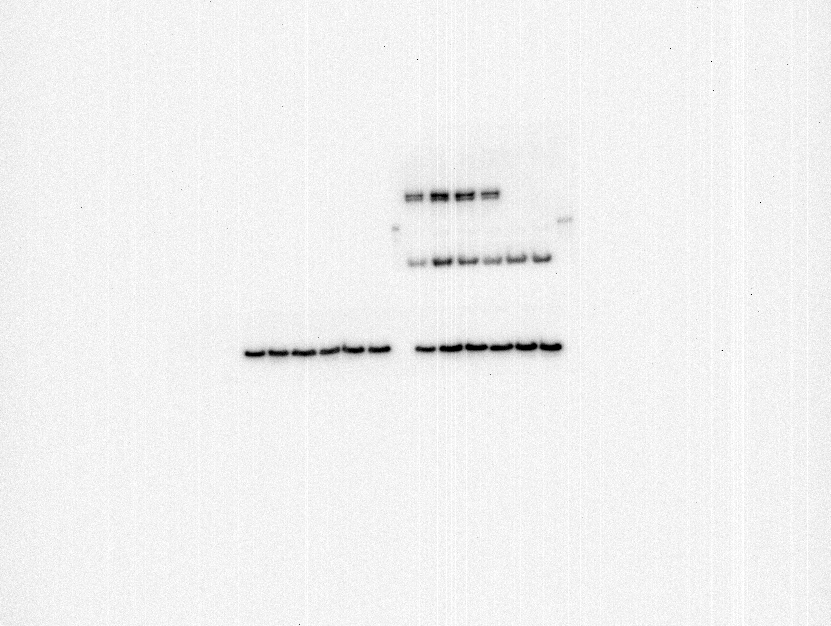

Supplement: Figure 6—figure supplement 1—source data 1. [file elife-94001-fig6-figsupp1-data1.zip › Figure 6-figure supplement 1 source data 2 Gapdh western .png]

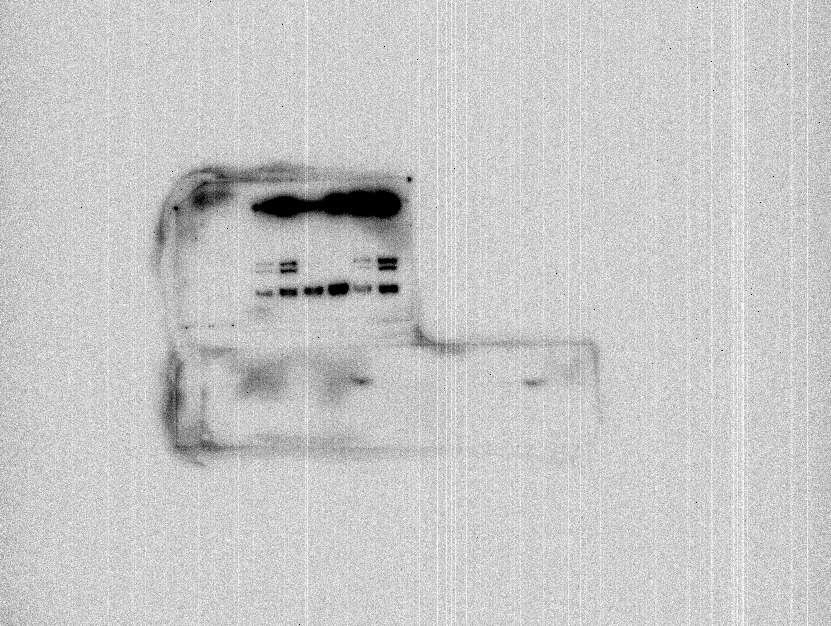

Supplement: Figure 6—figure supplement 1—source data 1. [file elife-94001-fig6-figsupp1-data1.zip › Figure 6-figure supplement 1 source data 2. Cul4B Cul4A westerns.png]

Figure 6-figure supplement 1 source data 2

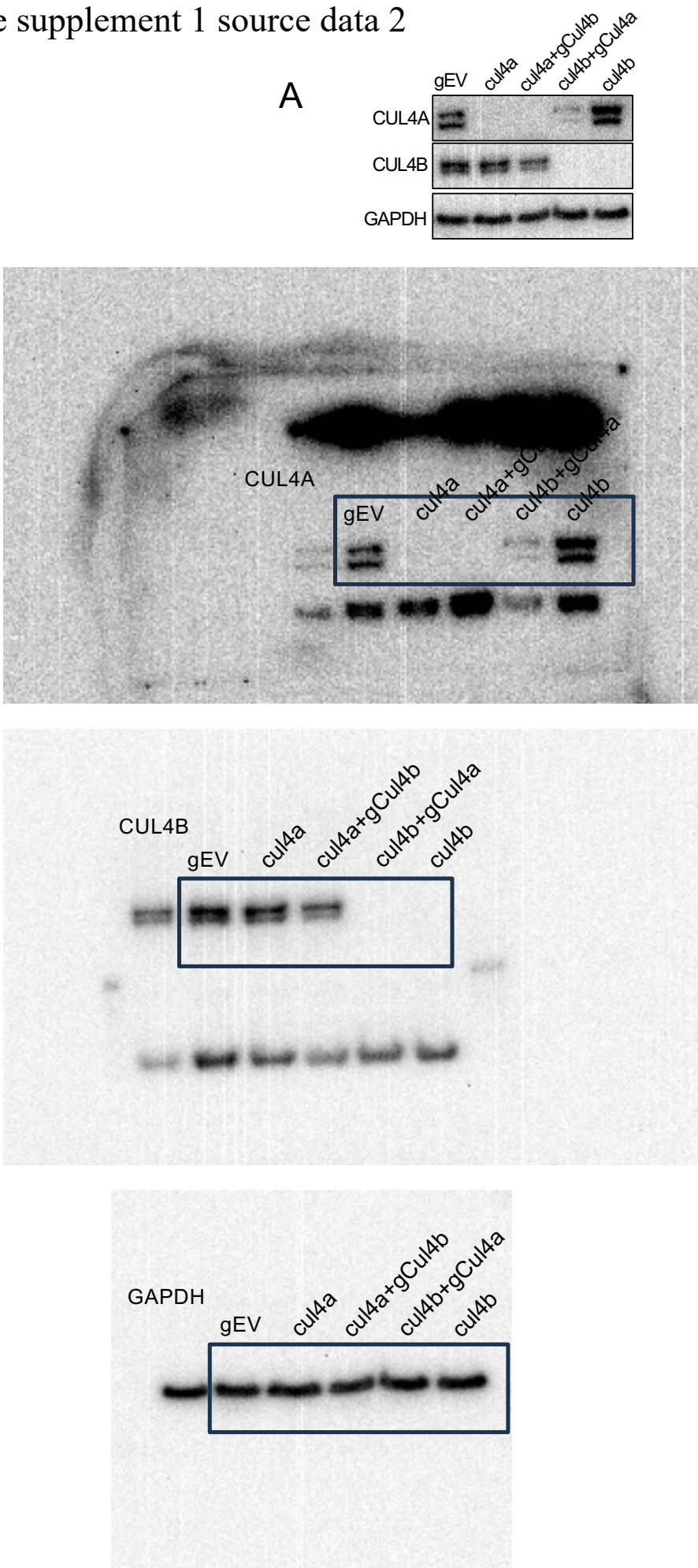

Supplement: Figure 6—figure supplement 1—source data 2. [file elife-94001-fig6-figsupp1-data2.pdf]
