# Supplementary material for: Transcriptional inhibition after irradiation occurs preferentially at highly expressed genes in a manner dependent on cell cycle progression
Source: eLife. 2024 Oct 11;13:RP94001. doi: 10.7554/eLife.94001 (PMC11469672; doi:10.7554/eLife.94001)

Figure 1-figure supplement 2 source data 4

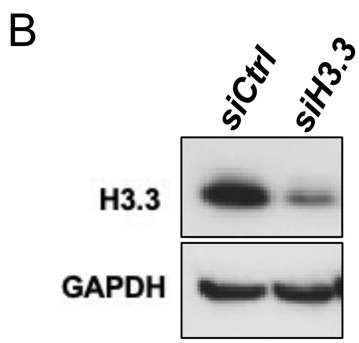

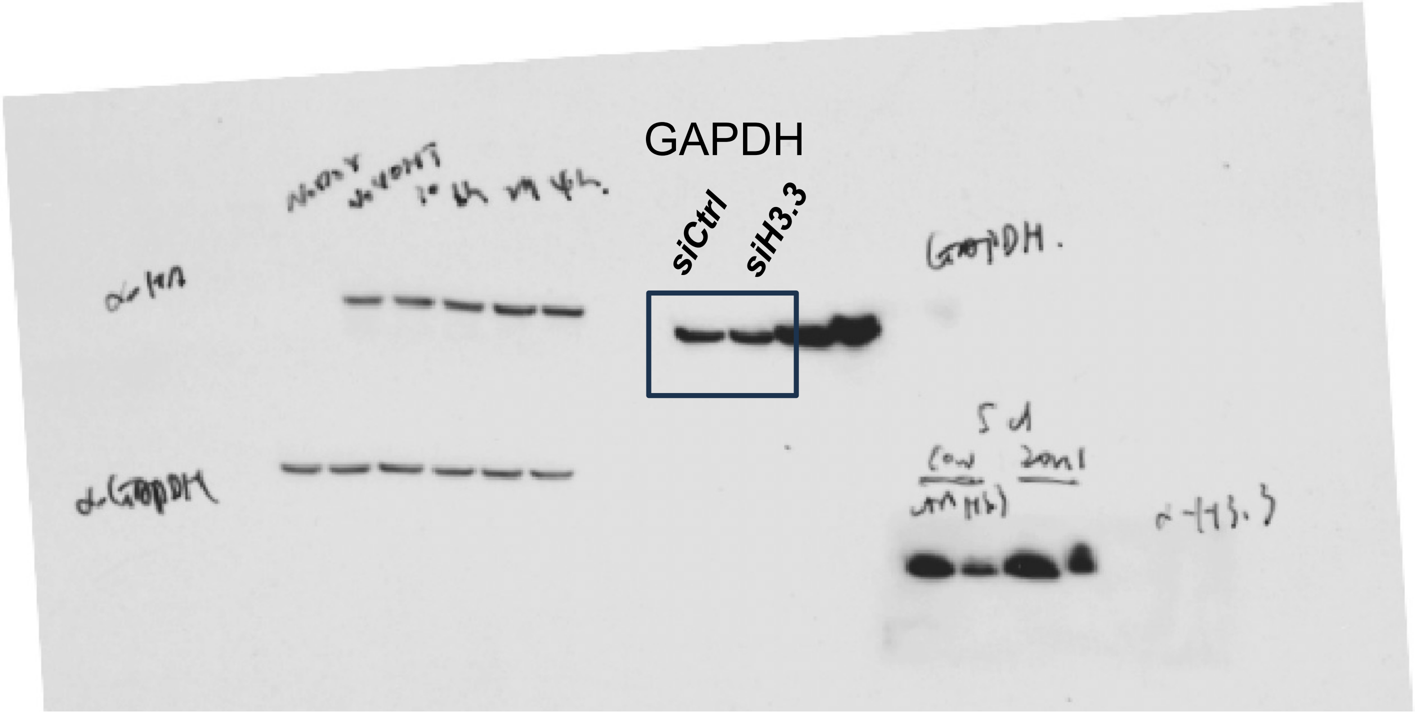

Image flipped from Figure 1-figure supplement 2 source data 3

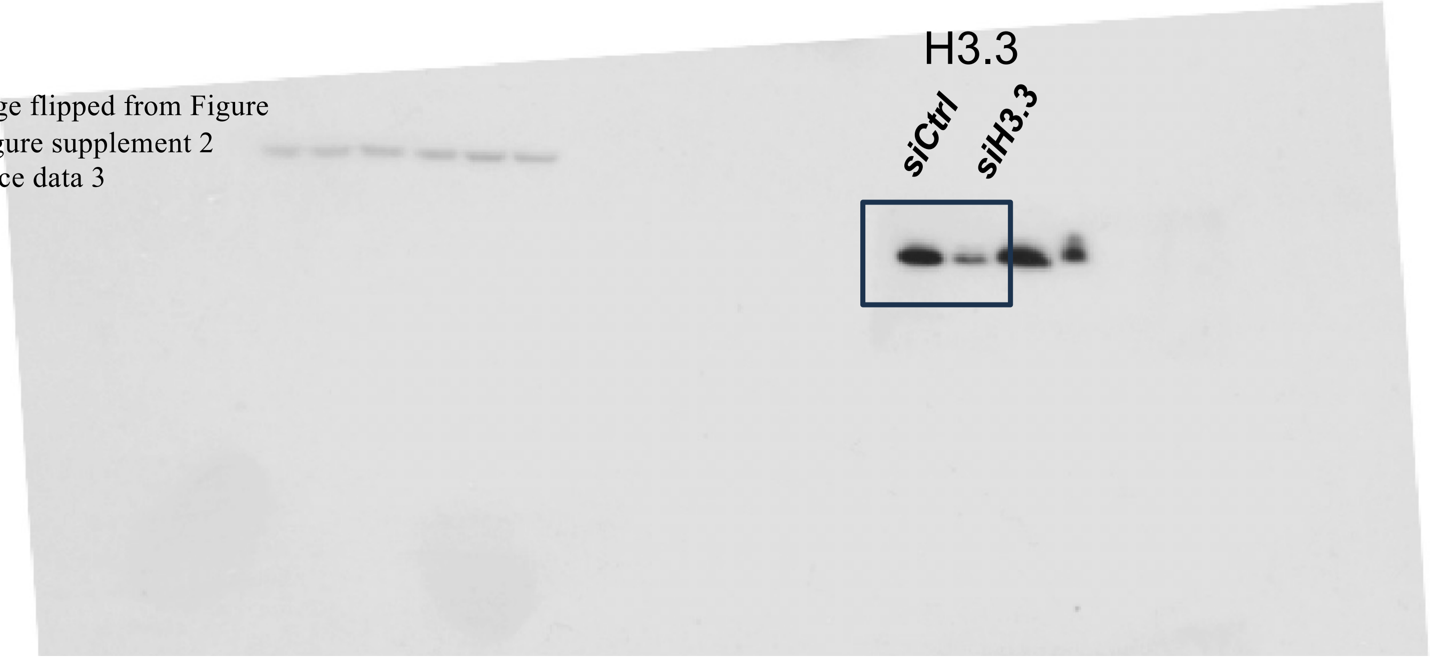

Supplement: Figure 1—figure supplement 2—source data 4. [file elife-94001-fig1-figsupp2-data4.pdf]
